# Reviewers in 2022

**DOI:** 10.1098/rsob.230060

**Published:** 2023-03-15

**Authors:** Jon Pines

**Affiliations:** Editor-in-Chief

The Editors of *Open Biology* would like to thank all reviewers who contributed their time by refereeing manuscripts submitted throughout 2022. From previous feedback, we know that most reviewers value recognition of their efforts by the journal and through services, such as Web of Science Reviewer Recognition Science, which is integrated with *Open Biology*.

At the start of 2023, we launched a new pilot scheme to reward peer review, where reviewers of *Open Biology* are awarded tokens that can be redeemed against article processing charges when they publish with us.

To further provide opportunities for recognition, we list the names of all reviewers last year who opted in. This article is made permanent and citeable by its digital object identifier (DOI). We encourage you to quote your contribution in your applications for tenure and grants or other forms of research assessment. Where you have provided it, we have included your ORCID, so your contribution can be unambiguously assigned to you.

The team really does appreciate all the work our reviewers do on behalf of the journal, and we look forward to working with you again in future.

Ahmad K https://orcid.org/0000-0002-1095-8445
Akiyoshi B https://orcid.org/0000-0001-6010-394X
Alberto-Silva C https://orcid.org/0000-0003-4519-8930
Alic N https://orcid.org/0000-0003-0356-6600
Allen J
Allingham J https://orcid.org/0000-0002-0918-3152
Andersson J
Antonicka H https://orcid.org/0000-0002-2525-2867
Arakawa K https://orcid.org/0000-0002-2893-4919
Archambault V https://orcid.org/0000-0002-2857-7667
Avella M
Ayté J
Badea TC
Baghel M
Ball E
Ballmer B
Bartoszewski R https://orcid.org/0000-0002-2864-6757
Bax BD
Becker D https://orcid.org/0000-0003-4315-8628
Bertolin G
Bhanot P
Bhartiya D
Bilandžija H
Blaydes JP
Bodas M
Brown M
Brownlee C
Bruce A https://orcid.org/0000-0003-4297-4412
Brunke S
Cao N
Capra V
Carmena A
Carvalho LP
Carver J
Casali A
Castanheira F
Centanin L
Chakravortty D
Chan KY https://orcid.org/0000-0002-5974-1650
Chen X
Chen L
Clowney J
Columbus L
Colvin R
Connock M
Corbet C
Coulson E
Cross R
Cuesta A
D'Adamo S
Dai Z
Das M
De Meyts P https://orcid.org/0000-0001-6214-0824
Devenport D https://orcid.org/0000-0002-5464-259X
Devineni A
Dey G https://orcid.org/0000-0003-1416-6223
Di Cristo G
Dickinson G
Dominguez I https://orcid.org/0000-0001-9617-4141
Domínguez-Pérez R https://orcid.org/0000-0001-8979-8394
Draskovic I
Duckett SK
Duncan E
Echeverri K
Elias M https://orcid.org/0000-0003-0066-6542
Ellender T
Elsayad K
Emme L
Enguita FJ
Evavold C
Feng T
Fisher R
Fraint E
Francia M https://orcid.org/0000-0001-9664-9573
Freeland S https://orcid.org/0000-0003-4606-7617
Fromm B
Funabiki H https://orcid.org/0000-0003-4831-4087
Ganz J
Gayathri P https://orcid.org/0000-0002-2242-4619
Ge L
Glasner ME https://orcid.org/0000-0003-1818-1965
Good M
Grau DP
Magdalena G
Gründer S
Guettler S
Han C
Hasan G
Hassan PZ
Herrera SC
Hill S
Houten SM
Hu S
Hubalek F
Ikui A
Ittner A
Iwai H
Jakubik J https://orcid.org/0000-0002-1737-1487
Jamuna Surendran S
Jauch R
Jessop M
Jia S
Jiang C
Jouandin P
Jovine L
Jr WW Hay
Kale V
Kanda T https://orcid.org/0000-0002-1391-3349
Kappen C
Karnkowska A
Karu K
Kawamukai M
Kessler T
Khan K
Kins S
Kitazawa T https://orcid.org/0000-0002-3377-493X
Knorre D https://orcid.org/0000-0001-7468-6963
Koonin E https://orcid.org/0000-0003-3943-8299
Korb J https://orcid.org/0000-0001-9577-9376
Kotak S
Kuil L
Kumamoto E
Kursula I https://orcid.org/0000-0001-5236-7056
Lanz Mendoza H
‪Lasker ‪K
Lee K
Li L
Li S
Li G https://orcid.org/0000-0002-0599-5356
Lilly B
Lin S
Linder M https://orcid.org/0000-0003-2202-9712
Liu M-F
Longdon B https://orcid.org/0000-0001-6936-1697
Madrid M
Malnic B
Mansur D
Martino S
Matsumoto S
Mazza D
Meuti M
Meyer K
Mikus F
Minli Y https://orcid.org/0000-0003-3373-8012
Mitchell B
Mitchell D
Mitsunaga-Nakatsubo K https://orcid.org/0000-0001-8307-9399
Möbius W
Moran Y https://orcid.org/0000-0001-9928-9294
Moreira J
Morrison C
Mosqueda N https://orcid.org/00000000238606759
Mudge J https://orcid.org/0000-0003-4789-7495
Muhi S
Nadler A
Nakabeppu Y
Nakajima Y https://orcid.org/0000-0002-1823-5989
Nasher F
Nathanson L https://orcid.org/0000-0003-1038-9083
Navarro Gil P
Offre P
Opresko P
Fabian P http://orcid.org/0000-0001-5359-4699
Penadés JR https://orcid.org/0000-0002-6439-5262
Pfeifer E
Pinotsis N
Poirot M https://orcid.org/0000-0002-5711-6624
Posern G https://orcid.org/0000-0002-0400-0222
Prinz A
Pugliese G
Rao H
Ravindran V
Roberts A
Rogers L https://orcid.org/0000-0002-9956-1769
Romero E
Roncarati D
Saenz J
Samways D
Sandoz JC https://orcid.org/0000-0002-5423-9645
Saunders T
Sazanov L
Schalch T
Scheele C https://orcid.org/0000-0001-8999-5451
Schreiber S
Schwartz GJ
Sdelci S https://orcid.org/0000-0003-1330-4364
Sharan R
Shi Z
Shibata N
Shu G
Siden-Kiamos I https://orcid.org/0000-0003-1572-5840
Sieben C
Silva J
Sintim H
Skittrall J https://orcid.org/0000-0002-8228-3758
Skupin A
Snel B
Soto M
Spletter ML
Stammnitz M https://orcid.org/0000-0002-1704-9199
Stark A https://orcid.org/0000-0002-4217-2850
Sucena É
Sunol C
Sunter J http://orcid.org/0000-0002-2836-9622
Swartz Z
Taniguchi K
Tao Y-X
Tasaki E https://orcid.org/0000-0002-6514-5134
Tennant D
Tennessen J
Tokatlidis K https://orcid.org/0000-0001-6295-8183
Tomanin R https://orcid.org/0000-0002-3346-9900
Tritten L
Ussar S
Vagnarelli P https://orcid.org/0000-0002-0000-2271
Van Aken O https://orcid.org/0000-0003-4024-968X
Vecsey CG
Vega M
Vincent B
Vogt K https://orcid.org/0000-0002-8763-342X
Wang Q
Wang X
Weijer C
Weng D
Wickstead B
Wille H
Wirtshafter H
wong wf https://orcid.org/0000-0003-1082-1956
Wood J
Xu F https://orcid.org/0000-0001-9965-9646
Xu H
Yamamoto D
Yang H
Yang J-S https://orcid.org/0000-0001-9910-3170
Zhang Y-A https://orcid.org/0000-0002-9956-3879
Zhang P
Zhao C
Zhong F
Zhu R

